# Dual targeting with ^224^Ra/^212^Pb-conjugates for targeted alpha therapy of disseminated cancers: A conceptual approach

**DOI:** 10.3389/fmed.2022.1051825

**Published:** 2023-01-17

**Authors:** Asta Juzeniene, Vilde Yuli Stenberg, Øyvind Sverre Bruland, Mona-Elisabeth Revheim, Roy Hartvig Larsen

**Affiliations:** ^1^Department of Radiation Biology, Institute for Cancer Research, The Norwegian Radium Hospital, Oslo University Hospital, Oslo, Norway; ^2^Department of Physics, University of Oslo, Oslo, Norway; ^3^Institute for Clinical Medicine, University of Oslo, Oslo, Norway; ^4^ARTBIO AS, Oslo, Norway; ^5^Department of Oncology, The Norwegian Radium Hospital, Oslo University Hospital, Oslo, Norway; ^6^Division of Radiology and Nuclear Medicine, Oslo University Hospital, Oslo, Norway

**Keywords:** cancer, lead-212, radiopharmaceutical, radium-224, radium-223, targeted radionuclide therapy (TRT), targeted alpha particle therapy (TAT)

## Abstract

Metastases are the primary cause of death among cancer patients and efficacious new treatments are sorely needed. Targeted alpha-emitting radiopharmaceuticals that are highly cytotoxic may fulfill this critical need. The focus of this paper is to describe and explore a novel technology that may improve the therapeutic effect of targeted alpha therapy by combining two radionuclides from the same decay chain in the same solution. We hypothesize that the dual targeting solution containing bone-seeking ^224^Ra and cell-directed complexes of progeny ^212^Pb is a promising approach to treat metastatic cancers with bone and soft tissue lesions as well as skeletal metastases of mixed lytic/osteoblastic nature. A novel liquid ^224^Ra/^212^Pb-generator for rapid preparation of a dual targeting solution is described. Cancer cell targeting monoclonal antibodies, their fragments, synthetic proteins or peptides can all be radiolabeled with ^212^Pb in the ^224^Ra-solution in transient equilibrium with daughter nuclides. Thus, ^224^Ra targets stromal elements in sclerotic bone metastases and ^212^Pb-chelated-conjugate targets tumor cells of metastatic prostate cancer or osteosarcoma. The dual targeting solution may also be explored to treat metastatic breast cancer or multiple myeloma after manipulation of bone metastases to a more osteoblastic phenotype by the use of bisphosphonates, denosumab, bortezomib or hormone therapy prior to treatment. This may improve targeting of bone-seeking ^224^Ra and render an augmented radiation dose deposited within metastases. Our preliminary preclinical studies provide conceptual evidence that the dual ^224^Ra-solution with bone or tumor-targeted delivery of ^212^Pb has potential to inhibit cancer metastases without significant toxicity. In some settings, the use of a booster dose of purified ^212^Pb-conjugate alone could be required to elevate the effect of this tumor cell directed component, if needed, e.g., in a fractionated treatment regimen, where the dual targeting solution will act as maintenance treatment.

## Introduction

Wide-spread (skeletal, lymph and/or visceral) metastases are responsible for ∼70% of cancer mortality worldwide (1, 2). Understanding and developing targeted therapies for metastatic cancers remain a large unmet medical need. Therapeutic nuclear medicine is emerging rapidly as an additional treatment modality in oncology (3–5). Recently approved beta-emitting ^177^Lu-DOTATATE (Lutathera^®^, 2018) targeting somatostatin-2 receptors in patients with metastatic neuroendocrine tumors and ^177^Lu-PSMA-617 (Pluvicto^®^, 2022) targeting prostate-specific membrane antigen (PSMA) in patients with metastatic castration-resistant prostate cancer (mCRPC) will clearly shift targeted radionuclide therapy (TRT) into the mainstream of cancer treatment. Nevertheless, some patients either do not respond or following initially good response develop resistance to ^177^Lu-based therapies, despite sufficient expression of target proteins (6, 7). These patients may, however, respond to targeted alpha therapy (TAT) with ^225^Ac (6, 7). Both preclinical and clinical studies have clearly demonstrated that alpha-emitting radiopharmaceuticals are more efficient in tumor cell killing and less damaging to the surrounding normal tissue than beta-emitting radiopharmaceuticals (5, 8–11). Alpha particles deliver a high amount of ionization over a short range (< 100 μm in water/tissue, < 40 μm in bone), inducing more complex double-strand DNA breaks that are harder to repair than single-strand breaks induced by beta particles (10, 11). Alpha-emitting radionuclides are particularly suited for the elimination of single cells and cancer micrometastases (10). TRT with beta- or alpha- emitting radionuclides improve the quality of life and delay disease progression (12, 13), but they are most likely not curative. Further improvements are warranted to enhance the therapeutic benefit. Combining TRT with potentially synergistic agents (chemotherapy, immune checkpoint inhibitors, PARP inhibitors, etc.) or with other radiopharmaceuticals is being evaluated in ongoing clinical trials (14–17). The focus of this paper is to describe and explore a novel technology platform that may improve the therapeutic effect of TRT by combining two radionuclides from the same decay chain; one TAT component targeting the stromal elements of osteoblastic skeletal metastases and the other by selective cell-surface binding to cancer cells in extraskeletal and skeletal metastases.

## Dual targeting strategies: Alpha and beta radiopharmaceuticals

It has been demonstrated that tandem therapy with beta-emitting ^177^Lu-PSMA-617 and alpha-emitting ^225^Ac-PSMA-617 is an effective treatment approach for mCRPC patients (18–20). In addition, the combination of ^177^Lu-PSMA-I&T and ^225^Ac-J591 for progressive mCRPC (33 patients) is being evaluated in an ongoing phase I/II clinical study in the United States (ClinicalTrials.gov Identifier: NCT04886986). It has been hypothesized that additive radiation to PSMA-positive cells should occur when administering the radiopharmaceuticals concurrently since the monoclonal antibody (mAb) J591 and the small molecule ligand PSMA-I&T have different PSMA binding sites (21). Additionally, the team hypothesized that ^225^Ac-J591 could deliver antitumor activity without xerostomia (21, 22), that is the most common side effect of PSMA-TAT with small molecule ligands (13). However, at the present time, the insufficient availability and radiopharmaceutical aspects of ^225^Ac limit the wide clinical applications of ^225^Ac (23–25).

The first, and so far, only approved alpha-emitting radiopharmaceutical ^223^RaCl_2_ (Xofigo^®^, 2013) is used to treat mCRPC that has spread only to the bone (3, 26). Ra-223 binds to osteoblastic stromal elements of bone metastases during mineralization since ^223^Ra is a calcium mimetic that binds to hydroxyapatite in the bone matrix in areas of high bone turnover. Such osteoblastic bone metastases are predominant in patients with mCRPC (27–29). The spatial distribution of the hydroxyapatite within an osteoblastic tumor facilitates a volume distribution of ^223^Ra (30). Due to the bone-seeking characteristics of ^223^Ra, its clinical use is limited to patients with osteoblastic bone (sclerotic, new bone deposition, or formation) metastases (31). Biologically stable complex between a bifunctional chelator with ^223^Ra and a tumor-targeting vector (small molecule, peptide, mAb, or its fragment) is essential to treat extraskeletal metastases (lymph nodes and visceral). Unfortunately, ^223^Ra, like other alkaline earth metals, does not form stable complexes *in vivo* (32–34). A phase I/II study, the AlphaBet trial, evaluating the combination of ^177^Lu-PSMA-I&T and ^223^Ra to target PSMA-expressing cancer cells and bone metastasis in 36 mCRPC patients has recently been started in Australia (NCT05383079).

## Dual targeting strategy: A cancer cell-surface seeker targeted ^227^Th and stromal bone-seeker ^223^Ra

**FIGURE 1:**
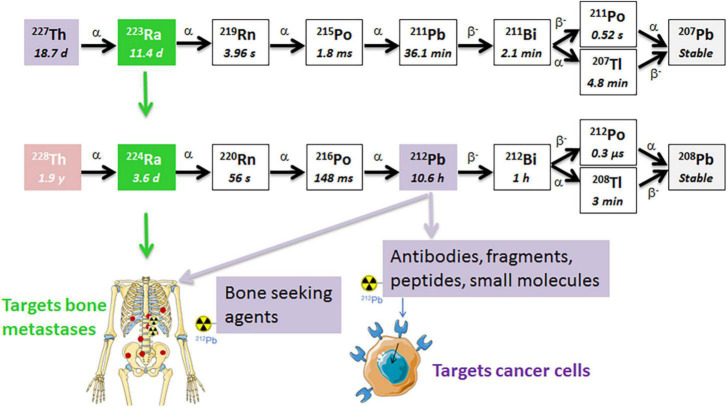
The decay chains of ^227^Th and ^228^Th. The radium isotopes are chemically similar to calcium and are natural bone-seekers, and thus, will target osteoblastic bone metastases. The progeny ^212^Pb in the ^224^Ra decay chain has a suitable half-life for chelation by a tumor-specific ligand/mAb that targets cancer cells (41, 54, 55). The short half-life of progeny ^211^Pb in the ^223^Ra decay chain is not practical for conjugation to a targeting ligand since the majority of the decay will occur before the ligand reaches the tumor sites. α, alpha particle; β, beta particle.

Another radionuclide that attracts interest is ^227^Th that can be linked to a variety of mAbs. These ^227^Th-immunoconjugates have shown promising preclinical results (30, 35, 36). Furthermore, ^227^Th acts as an *in vivo* generator of bone-seeking ^223^Ra (Figure 1) that can be additionally exploited to improve therapeutic effects in sclerotic bone metastases (30, 37). Dual bone-targeting strategy by bone-targeted ^227^Th and ^223^Ra was introduced in 2004 (38, 39). Radium-223 produced from ^227^Th decay is a cation that can easily penetrate into sclerotic metastasis. Henriksen et al. suggested to use ^227^Th-polyphosphonate compounds, DOTMP [1,4,7,10 tetraazacyclododecane N, N′, N″, N″ 1,4,7,10-tetra(methylene) phosphonic acid] or DTMP [diethylene triamine N, N′, N″ penta(methylene) phosphonic acid] to deliver alpha particle radiation to primary bone cancer or skeletal metastases from solid cancers (38). They proposed that the total radioactivity in bone should increase as ^227^Th decays and ^223^Ra appears, if the ^227^Th–labeled bone-seeker solution was free from ^223^Ra at the time of administration (38). Washiyama et al. studied the biodistribution of bone-seeking ^227^Th-EDTMP (ethylenediamine-tetramethylenephosphonic acid) and its daughter ^223^Ra in mice and found high uptake of ^227^Th-EDTMP and long retention of ^223^Ra in bones (39). They concluded that even if ^223^Ra escapes from the bone after ^227^Th decay, it redistributes to bone, as there are no physical or biological differences between ^223^Ra injected intravenously and that generated *in vivo* after ^227^Th injection (39). In 2008, a dual targeting approach was introduced for the treatment of soft tissue and bone metastases: ^227^Th-chelator-mAb targeting cancer cell surface antigens and ^223^Ra targeting osteoblastic stroma (30, 37). The main advantage of ^227^Th is high availability from beta decay of ^227^Ac (36). The 18.7 day half-life of ^227^Th is long enough for proper radiopharmaceutical preparation, transportation and administration. However, a therapeutic window allowing treatment with ^227^Th-conjugates with acceptable toxicity may exist due to ^223^Ra ingrowth. A few clinical trials evaluating ^227^Th-conjugates targeting CD22, mesothelin, PSMA and human epidermal growth factor 2 (HER-2) are registered at clinicaltrials.gov (NCT02581878, NCT03507452, NCT03724747, NCT04147819), but the results are not yet available.

## Dual targeting strategy: A bone-seeker ^224^Ra and a cancer cell-seeker targeted ^212^Pb in one solution

Two radionuclides from the same decay chain, ^224^Ra and ^212^Pb, with its alpha emitting daughter ^212^Bi (Figure 1), are attractive for cancer therapy due to availability and physical half-lives. These radionuclides can be produced by the end user in clinically relevant amounts from ^228^Th generators (40–42). The 1.9 year half-life of ^228^Th allows long-term use of the generator. Similarly to ^223^Ra, the lack of suitable chelators has limited ^224^Ra to bone-targeting applications. Both radium isotopes have similar chemical and decay properties, total energies, and biodistribution (41, 43–45). Importantly, ^224^Ra was widely used decades ago as a pain-relieving treatment of the chronic inflammatory rheumatic disease ankylosing spondylitis (46–49). Administration of total activities of 5.6–11.1 MBq ^224^Ra (1 MBq of ^224^Ra chloride solution per weekly injection) to these patients between 1945 and 1975 had neither negative impact on the survival, nor increased significantly the overall rate of second malignancies, as compared to the control population after a mean follow-up time of 24 years (46). The incidence rates of leukemias were 0.014 and 0.009 [hazard ratio 2.56 (95% confidence interval (CI) 0.89–7.54)] in in patients treated and non-treated with ^224^Ra, respectively (46, 48, 50). Such long-term follow up of a large non-cancer patient population seems very relevant from a radiation safety perspective. The life expectancies in patients with metastatic cancer are significantly shorter.

**FIGURE 2:**
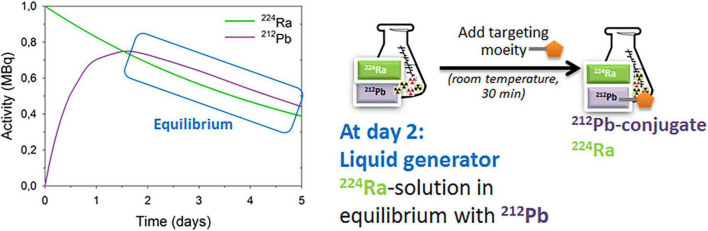
The ^224^Ra-liquid generator for preparation of dual alpha targeting solution. For details see references (41, 54, 55).

The daughter nuclides of ^224^Ra, namely ^220^Rn (t_1/2_ ≈ 56 s), ^212^Pb (t_1/2_ ≈ 10.6 h), and ^212^Bi (t_1/2_ ≈ 1 h), have longer half-lives than those of ^223^Ra (Figure 1). Lead-212 is suitable for radiolabeling of mAbs, peptides, or other targeting vectors conjugated with appropriate bifunctional chelators. Conjugates of ^212^Pb/^212^Bi have already been tested in clinical trials for cancer treatment (51–53). Moreover, these daughter nuclides can be conjugated to chelated targeting agents in the radiopharmaceutical solution of ^224^Ra in equilibrium with progeny (Figure 2), such as EDTMP for retention of progeny in bone, or a cancer-specific ligand/mAb with a bifunctional chelator TCMC (S-2-(4-isothiocyanatobenzyl)-1,4,7,10-tetraaza-1,4,7,10-tetra(2-carbamoylmethyl)cyclododecane) for cancer cell targeting (41, 54, 55). We hypothesize that the resulting solution will have dual TAT properties: (1) Unbound bone-seeking ^224^Ra will target metastatic cells on the endosteal surface of bone as well as the stromal elements of osteoblastic skeletal metastases killing these cells; and (2) tumor cell-surface seeking ^212^Pb-TCMC-targeting agent that will kill the circulating cancer cells and micrometastases by selective binding and deposition of DNA breaking alpha radiation to the cancer cells. The aim of the dual targeting approach is to direct as much as possible of the ionizing radiation of the ^224^Ra decay chain to the entire spectrum of metastases.

## Dual targeting technology: The ^224^Ra/^212^Pb liquid generator

A ^224^Ra-liquid generator for the preparation of dual targeting solution (Figure 2) was developed and patented by Larsen (54). Targeting moieties can be rapidly (≤ 1 h) and efficiently labeled with ^212^Pb in the ^224^Ra-solution in transient equilibrium with progenies (41, 55). Additionally, up to 80% free ^212^Bi can also be conjugated (41). Similar binding and uptake abilities of the ^212^Pb-labeled PSMA-targeting ligand NG001 in ^224^Ra-solution or in ^212^Pb-solution were observed *in vitro* and *in vivo* (41, 56, 57). Biodistribution studies of ^224^Ra with free ^212^Pb, ^212^Pb-NG001, and ^212^Pb-PSMA-617 were tested in athymic nude mice with C4-2 xenografts (41). Importantly, a high uptake of ^224^Ra in the femur and skull in all groups was shown, demonstrating that ^212^Pb can be chelated to ligands without compromising the bone-seeking properties of radium in the radiopharmaceutical solution containing the radionuclides (41).

## Diffusion of ^224^Ra progenies and “dose-smoothening effect”

As mentioned above, ^224^Ra has longer lived progenies, ^220^Rn and ^212^Pb, than the isotopes of the same elements in the ^223^Ra series (Figure 1). Theoretically, these daughter nuclides, especially noble gas ^220^Rn, will diffuse from the target, because of differing physical half-lives and biological affinities. The first ^220^Rn progeny, ^216^Po, has a half-life of 148 ms and decays within very close vicinity to the creation site (58). The mean diffusion length of ^216^Po in water (soft tissue) is only around 4 μm (58). Lloyd et al. studied retention and distribution of ^224^Ra and its daughters in beagle dogs, and concluded that the majority of ^220^Rn produced in bone by ^224^Ra decay stays in bone (59). It has been demonstrated that ^220^Rn redistribution leads to toxicity to non-targeted tissues only when extremely high activities of ^224^Ra were given to patients or animals (47, 59–61). Napoli et al. studied the diffusion and re-adsorption of ^224^Ra progenies from ^224^Ra-labeled calcium carbonate microparticles (61). These particles were chosen since radium’s calcium-mimetic properties allow the adsorption of ^224^Ra onto their surface. It has been demonstrated that ^220^Rn can escape from the particles, however, it can diffuse from ^224^Ra-labeled calcium carbonate microparticles only around 300–400 μm in water. It was also documented that the microparticles have the ability to re-adsorb almost all ^212^Pb generated in the liquid phase from escaped ^220^Rn (61). If we assume that these particles resemble bone or an osteoblastic bone metastasis, the obtained results may explain the low leakage of ^212^Pb from bone into systemic circulation, thereby reducing the risk of unwanted radiation exposure of distant tissues. The diffusion of ^220^Rn up to a few hundred micrometers can extend the effective range of the shorter-range alpha particles from ^224^Ra itself and may cause a “dose-smoothening” effect in the metastases. Ra-223 is initially shown to be deposited on bone surface and with time is incorporated into the volume of the bone (59). However, areas with “uncalcifying” stroma containing cancer cells in bone metastases (29) will most likely not be eradicated. This contribution from ^220^Rn may overcome one major limitation of the approved bone-seeking radiopharmaceutical Xofigo when it comes to the short range (only up to 40 μm in bone) of alpha particles in skeletal metastases.

Diffusing alpha-emitters radiation therapy (DaRT) is a novel brachytherapy employing implantable ^224^Ra enriched seeds for the treatment of solid tumors (58, 62, 63). The ^224^Ra progenies are shown to diffuse 5–7 mm from the seed and are reported as responsible for the therapeutic effect (63). The efficacy and safety of DaRT have been found to be promising in preclinical and clinical studies (62, 63). DaRT is now in clinical trials for many different cancer types (Table 1).

**TABLE 1:** List of ongoing clinical trials with diffusing alpha-emitters radiation therapy (DaRT).

Cancer	Clinical study identifier
Squamous cell carcinoma (SCC)	NCT03353077, NCT05065346, NCT05047094, NCT04068155
Cutaneous, Mucosal, Superficial Soft Tissue Neoplasia	NCT03737734, NCT03886181, NCT03889899, NCT04534127, NCT04540588
Prostate	NCT04543903
Breast	NCT03970967, NCT04906070
Pancreatic	NCT04002479
Vulva	NCT04761146

As mentioned above three of the four alpha particles in the decay chain of ^224^Ra will decay within a radius of about 400 μm from the ^224^Ra atom in tissues, while the ^212^Pb due to the long half-life can potentially diffuse up to several mm in tissues as indicated by the DART technology. If decay takes place in the skeleton, association of ^212^Pb to hydroxyapatite may considerably limit diffusion range.

## Dual targeting alpha therapy and cancers with bone metastases

Bone is the third most frequent site of cancer metastases and the organ-system involved in multiple myeloma (64–66). Bone metastases are especially common in prostate and breast cancer (Table 2). These metastases frequently result in skeletal-related events such as increased pain, hypercalcemia, bone fractures and spinal cord compression, which cause considerable morbidity and reduced quality of life (1, 66, 67). Bone metastases occur as osteolytic lesions, characterized by destruction of normal bone, or as osteoblastic metastases, characterized by formation of new bone matrix (Table 2). The majority of patients with advanced prostate cancer have osteoblastic bone metastases (29). However, some of these patients may have a mixed phenotype or even osteolytic lesions (29).

**TABLE 2:** Incidence of bone metastases in advanced cancer.

Primary cancer	Incidence of bone metastases (%)	Dominant type of bone metastases	Frequency of skeletal-related events (%)	References
Prostate	65–85	Osteoblastic	49	(64, 66, 68)
Breast	65–75	Mixed osteolytic/osteoblastic	64–68	(64, 66, 68)
Multiple myeloma	80–90	Osteolytic	51	(68–70)
Renal	20–40	Osteolytic	34	(64, 67, 68)
Lung (non-small cell)	30–60	Osteolytic	60	(64, 71, 72)
Lung (small cell)	34–50	Osteoblastic	9–63	(64, 66, 73, 74)
Neuroendocrine tumors	15–21	Mixed osteoblastic/osteolytic	26	(75)
Bone cancers (osteosarcoma)	Bone cancer	Osteoblastic	100%	(76)

Dual targeting alpha therapy seems the most suitable for prostate cancer and osteosarcoma since radium localizes in osteoblastic active zones, including on skeletal surfaces and in osteoblastic metastases (77, 78). For cancers without extraskeletal metastases, ^212^Pb can be chelated to organic phosphates, e.g., EDTMP, which are incorporated into the bone matrix (79), whereas for cancers with extraskeletal metastases, ^212^Pb can be chelated to small molecules or mAbs targeting cancer cells.

## Stromal manipulations: From osteolytic to osteoblastic

The skeletal lesions in multiple myeloma, breast, renal, and lung cancer patients are most commonly osteolytic (Table 2). Additionally, patients with these cancers may have extraskeletal metastases. A few clinical trials are registered to explore the potential of ^223^Ra, mainly in combination with other drugs (Table 3). However, bisphosphonates, denosumab, bortezomib, and antihormonal therapies may alter the bone matrix of the disease and lead to a more avid target for radium (80, 81). It is documented that the administration of bisphosphonates alters the lytic/blastic ratio in bone lesions toward a more blastic phenotype, and increase uptake of bone-seeking beta-emitting radiopharmaceuticals, such as ^89^Sr and ^153^Sm-EDTMP (81). Bortezomib and other proteasome inhibitors can also restore the impaired osteoblast activity (81–83). Denosumab is a mAb that binds the cytokine receptor activator of NFκB ligand (RANKL) that is an essential factor initiating bone turnover (84). RANKL inhibition blocks osteoclast maturation, function and survival, thus reducing bone resorption (84). It has been demonstrated that breast cancer patients after chronic bisphosphonate therapy and multiple myeloma patients after bortezomib treatment had increased ^99^Tm-labeled methylene diphosphonate (MTD) uptake in osseous bone metastases (81).

**TABLE 3:** List of clinical trials with Xofigo alone or in combination with other drugs in cancers with dominant osteolytic lesions.

Cancer	Drugs	Clinical study identifier
Relapsed multiple myeloma	Bortezomib, dexamethasone	NCT02605356, NCT02928029
Renal cell carcinoma	Pazopanib, sorafenib	NCT02406521
	Cabozantinib S-malate	NCT04071223
Breast	–	NCT01070485
	Exemestane, everolimus	NCT02258451
	–	NCT02258464
	Denosumab	NCT02366130
	Paclitaxel	NCT04090398
Lung	Pembrolizumab	NCT03996473
	–	NCT02283749

Ra-223 has been used for breast cancer patients with bone-dominant disease with osteoblastic and osteolytic lesions (85–87). Coleman et al. have demonstrated that ^223^Ra targeted osteoblastic, but not osteolytic lesions, in breast cancer patients with bone-dominant disease (85). The results are not surprising because the balance between osteoblastic and osteolytic lesions have not been taken into account (87). However, these studies have demonstrated that ^223^Ra is safe (85–87), with the potential to be combined with other therapies after pretreatment with bisphosphonates and denosumab.

Suominen et al. investigated the effect of ^223^Ra, bortezomib and their combination in the syngeneic 5TGM1 mouse multiple myeloma model *in vivo* (82). The combination of bortezomib and ^223^Ra improved the incorporation of ^223^Ra into multiple myeloma bone lesions, decreased synergistically the area of osteolytic lesions and decreased tumor burden and restored body weights in mice (82).

## Preclinical studies of dual targeting alpha therapy

Dual targeting alpha therapy seems more suitable for breast cancer patients than ^223^Ra alone because a ^212^Pb-conjugate potentially can target breast cancer cells all over the body or alternatively be made bone directed (i.e., dual bone targeting). Preclinical results demonstrated that a single dose of dual bone ^224^Ra-solution with EDTMP prolonged survival time and lowered incidence of paralysis and bone metastases in nude mice with breast cancer micrometastases (55). Epidermal growth-factor receptor (EGFR) is overexpressed in 15–70% of breast cancer (88, 89), and thus, is an attractive candidate for dual targeting alpha therapy.

Example 1

To test the proof of concept of our dual targeting approach the EGFR-targeting mAb cetuximab (CTX) and bone-targeting EDTMP were chosen for our pilot studies (unpublished results). The mAb or EDTMP were labeled with ^212^Pb in ^224^Ra solutions in equilibrium with progenies (pH adjusted to 5–6 by 0.5 M C_2_H_7_NO_2_ or C_2_H_3_NaO_2_). TCMC-mAb was added to a final concentration of 0.1–1 mg/ml. The solutions were mixed on a Thermomixer (Eppendorf, Hamburg) for 30 min at 37^°^C. Radiochemical purity of the samples was determined by instant thin layer chromatography (Tec-control, Biodex, Medical Systems, Shirley, NY), and only products with purities ≥ 95% were used in the experiments.

The anti-cancer effects of ^224^Ra-solutions with TCMC-CTX or EDTMP were investigated in 6 weeks old female athymic Nude-Foxn1nu mice (bred at the Comparative Medicine Department, Oslo University Hospital) with breast cancer metastasis. MDA-MB-231-luciferase (Luc) expressing breast cancer cells (2 × 10^5^ cells/100 μl PBS per mouse) were injected into the left ventricle of mouse heart (intracardiac injection). Sodium chloride (0.9% NaCl, control), 300 kBq/kg ^224^Ra & ^212^Pb-EDTMP, or 300 kBq/kg ^224^Ra&^212^Pb-TCMC-CTX were intravenously administered to mice 2 days after cell injection. Tumor metastases were monitored by bioluminescence imaging in an IVIS Spectrum *in vivo* imaging system (PerkinElmer, Waltham, MA) 24, 31, and 38 days after intravenous injection of compounds. Each mouse was injected intraperitoneally with 0.2 ml D-luciferin (Biosynth AG, Staad, Switzerland) dissolved in Dulbecco’s PBS (20 mg/ml) 10 min prior to imaging. During imaging, mice were under gas anesthesia (∼3.5% Sevoflurane in oxygen at 0.5 L/min; Baxter, IL, USA). All bioluminescence data are displayed in radiance (photons/s/cm^2^/str) under identical acquisition conditions. Mice were euthanized by cervical dislocation when cachexia, paraplegia or any signs of severe sickness or discomfort was observed. The studies were approved by the Institutional Committee on Research Animal Care (Department of Comparative Medicine, Oslo University Hospital) and the Norwegian Food Safety Authority (Brumunddal, Norway, approval: FOTS ID 22197).

**FIGURE 3:**
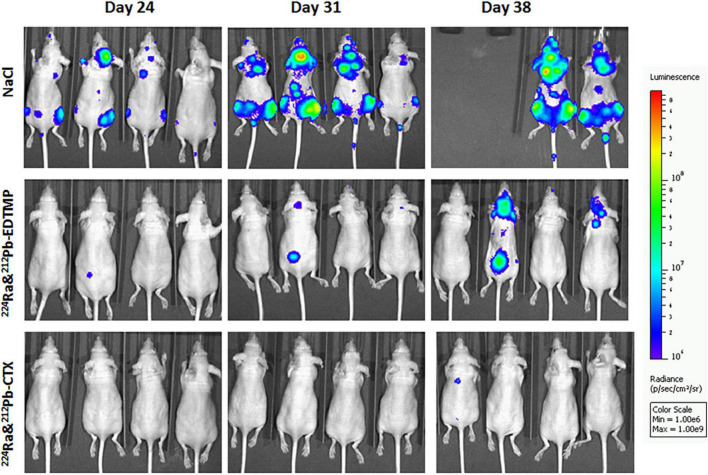
The influence of dual targeting alpha solution on breast cancer metastases growth in mice. Tumor metastases were monitored by bioluminescence imaging in the different therapy groups 24, 31, and 38 days after intravenous injection of 0.9% sodium chloride (NaCl, control), 300 kBq/kg ^224^Ra&^212^Pb-EDTMP, or 300 kBq/kg ^224^Ra&^212^Pb-TCMC-cetuximab (CTX). MDA-MB-231-Luc breast cancer cells (2 × 10^5^ cells/mouse) were injected intracardially into athymic Nude-Foxn1nu mice 2 days before the treatment. The mice are positioned in the same order at all-time points. The studies were approved by the Institutional Committee on Research Animal Care (Department of Comparative Medicine, Oslo University Hospital) and the Norwegian Food Safety Authority (Brumunddal, Norway, approval: FOTS ID 22197).

Dual targeting alpha therapy extended survival in EDTMP and cetuximab group compared to the control [0.9% sodium chloride (NaCl)] group, and lowered the incidence of bone and extraskeletal metastases (Figure 3). The preliminary studies provide conceptual and strong evidence that dual targeting ^224^Ra-solution with bone or tumor-targeted delivery of ^212^Pb has potential to inhibit cancer metastases without significant toxicity. Several molecular targets are being explored to target HER2, estrogen receptor and progesterone receptor for nuclear medicine imaging (90, 91), and they can be suitable for dual targeting alpha therapy of breast cancer after stromal manipulation.

Example 2

In prostate cancers, EGFR is weakly expressed in neoplastic cells while it is highly expressed in metastatic lesions (92, 93). The effectiveness of ^224^Ra-solution with ^212^Pb-TCMC-CTX directed against EGFR-positive multicellular LNCaP spheroids, an *in vitro* model for micrometastatic cancer, was investigated (unpublished data).

**FIGURE 4:**
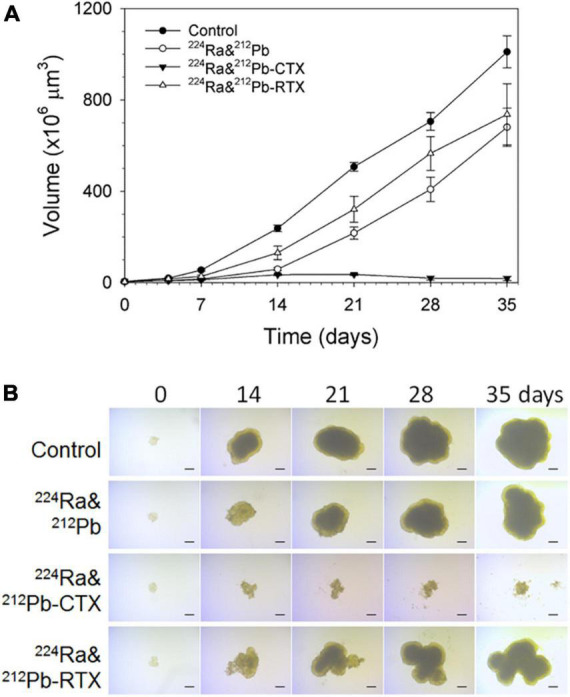
The influence of dual targeting alpha solution on prostate cancer multicellular LNCaP spheroid growth after incubation for 4 h. **(A)** Growth of spheroids treated with 1 kBq/ml ^224^Ra&^212^Pb, ^224^Ra&^212^Pb-TCMC-cetuximab (CTX), and ^224^Ra&^212^Pb-TCMC- rituximab (RTX, negative control) groups was measured for up to 35 days and is presented as volume (×10^6^ μm^3^) ± *SD*. The images of LNCaP spheroids were measured for up to 35 days. **(B)** Representative microscope images (×4 magnification) were taken at the predefined study end point of 35 days using a bright-field microscope with AxioVision Rel. 4.8 software. LNCaP spheroids were generated by cultivation of cells in liquid overlay in 1.5% agarose-coated flat bottom 96-well plates (94). Cell suspensions of 500 cells in 100 μL medium were added to each well, followed by centrifugation of the plates at 470 × g for 15 min. After an initial incubation time of 3 days, spheroids with diameter of ∼229 μm were formed.

Ra-224-solution with CTX effectively stopped the growth of LNCaP spheroids relative to the equivalent dose of ^224^Ra-solution alone or RTX (unpublished data, Figure 4).

## Transferring dual targeting solution into clinic

Several combination treatments with TAT have been proposed (95–98). Their goal is to increase efficacy by using therapies with different action mechanisms together with TAT keeping toxic effects to a minimum. Dual targeting solution may allow increasing therapeutic efficacy and reducing toxicities of ^224^Ra and its progenies to normal organs. Transferring dual targeting solution into clinic will be more complicated due to the complexity of the product (^224^Ra, its progenies, targeting agent, and chelator). A few therapy cycles of dual targeting alpha therapy, similarly to single alpha therapies, will be needed. In some cases, a booster dose of cancer cell-seeker targeted ^212^Pb alone will be required. At the same time, the non-toxic administered activity of ^224^Ra and/or ^212^Pb may be chosen based on earlier or ongoing clinical studies. As was mentioned earlier, ^224^Ra was used in the treatment of ankylosing spondylitis, and long-term toxicity and carcinogenicity data in humans exist (46–48). Additionally, the seeds with ^224^Ra (Table 1) and bio-degradable calcium carbonate microparticles with ^224^Ra (NCT03732768) are under clinical investigations for cancer treatment. A few clinical trials investigating ^212^Pb-conjugates are registered on Clinicaltrials.gov (Table 4). Phase I studies of ^212^Pb-TCMC-trastuzumab and ^212^Pb-DOTAMTATE demonstrated safety and feasibility in patients with HER-2 expressing malignancies (99) and somatostatin receptor (SSTR) expressing neuroendocrine tumors (52).

**TABLE 4:** List of clinical trials with ^212^Pb-conjugates.

Phase	Disease	Target	^212^Pb-conjugate	Clinical study identifier
1	Breast, peritoneal, ovarian, pancreatic and stomach neoplasms	Human epidermal growth factor 2 receptor (HER-2)	TCMC-trastuzumab	NCT01384253
1	Neuroendocrine tumor	Somatostatin receptor (SSTR)	DOTAMTATE	NCT03466216
2	Neuroendocrine tumor	Somatostatin receptor (SSTR)	DOTAMTATE	NCT05153772
1	Cutaneous melanoma, cervical, prostate, breast and colon cancers	Gastrin-releasing peptide receptor (GRPR)	DOTAM-GRPR1	NCT05283330

## Summary of dual targeting technology

The dual targeting solutions taking advantage of both the bone seeking ^224^Ra and cell directed complexes of ^212^Pb seems a promising approach to treat metastatic cancers presenting with bone and soft tissue lesions and also of skeletal metastases of mixed lytic/osteogenic nature. The radioactivity of the solutions will probably be dictated by the tolerability to the longer lived ^224^Ra. In this regard, the knowledge of long-term and short-term toxicity of ^224^Ra in the previous mentioned ankylosing spondylitis series may be important in determining the suitable activity levels. In some settings the use of a booster dose of purified ^212^Pb-radioligand alone could be a possible tactic to elevate the effect of this component, if needed, e.g., in fractionated scheduled treatment regimen, where the dual targeting solution then will act as maintenance treatment. It could be a regulatory challenge to develop such a combined product. Anyhow, some clinical data of the purified ^212^Pb-ligand alone would be required.

## Cell lines

The cell lines present in this study were obtained from ATCC (LNCaP cell line) and Cell Biolabs Inc. (MDA-MB-231-Luc cell line).

## Data availability statement

The raw data supporting the conclusions of this article will be made available by the authors, without undue reservation.

## Ethics statement

The animal study was reviewed and approved by the Norwegian Food Safety Authority (Brumunddal, Norway, approval: FOTS ID 22197).

## Author contributions

AJ, ØB, and RL: conceptualization. AJ, VS, ØB, and RL: designing the work and interpretation of results. AJ and RL: *in vitro, in vivo* experiments, and analyzing data. AJ, VS, ØB, M-ER, and RL: drafting the manuscript and revising it critically for important intellectual content. All authors have read and agreed to the published version of the manuscript.
